# Impact of function-guided glioma treatment on oncological outcome in the elderly

**DOI:** 10.1016/j.bas.2023.102742

**Published:** 2024-01-03

**Authors:** Carolin Albrecht, Lea Baumgart, Axel Schroeder, Benedikt Wiestler, Bernhard Meyer, Sandro M. Krieg, Sebastian Ille

**Affiliations:** a Department of Neurosurgery, Technical University of Munich, Germany; b Section of Diagnostic and Interventional Neuroradiology Department of Radiology, Klinikum Rechts der Isar, School of Medicine, Technische Universität München, Germany; c School of Medicine, Klinikum Rechts der Isar, Ismaninger Str. 22, 81675, Munich, Germany; d Department of Diagnostic and Interventional Neuroradiology, Technical University of Munich, Germany

**Keywords:** Elderly, Glioblastoma, Mapping, Neuromonitoring, Tms

## Abstract

**Introduction:**

Many patients with high-grade gliomas (HGG) are of older age.

**Research question:**

We hypothesize that pre- and intraoperative mapping and monitoring preserve functional status in elderly patients while gross total resection (GTR) is the aim, resulting in overall survival (OS) rates comparable to the general population with HGG.

**Material and methods:**

We subdivided a prospective cohort of 168 patients above 65 years with eloquent high-grade gliomas into four groups ([years/cases] 1: 65–69/58; 2: 70–74/47; 3: 75–79/43; 4: >79/20). All patients underwent preoperative noninvasive mapping, which was also used for decision-making, intraoperative neuromonitoring in 138 cases, direct cortical and/or subcortical motor mapping in 66 and 50 cases, and awake language mapping in 11 cases.

**Results:**

GTR and subtotal resection (STR) could be achieved in 65% and 28%, respectively. Stereotactic biopsy was performed in 8% of cases. Postoperatively, we found transient and permanent functional deficits in 13% and 11% of cases. Postoperative Karnofsky Performance Scale (KPS) did not differ between subgroups. Patients with long-term follow-up (51%) had a progression-free survival of 5.5 (1–47) months and an overall survival of 10.5 (0–86) months.

**Discussion and conclusion:**

The interdisciplinary glioma treatment in the elderly is less age-dependent but must be adjusted to the functional status. Function-guided surgical resections could be performed as usual, with maximal tumor resection being the primary goal. However, less network capacity in the elderly to compensate for deficits might cause higher rates of permanent deficits in this group of patients with more fast-growing malignant gliomas.

## Highlights

• Patients with gross total resection showed significantly longer survival than those with subtotal resection or biopsy
• Gross total resection is the primary goal for higher-grade gliomas, regardless of age, but dependent on functional status
• Non-invasive brain mapping enables the preservation of functionality, as shown by Karnofsky Performance Status

## 1 Introduction

Multimodal treatment of malignant glioma patients usually includes surgical resection, adjuvant radiotherapy, and chemotherapy. The EOR is one of the first factors contributing to the outcome as maximal tumor resection is considered a favorable prognostic factor, and studies have shown that surgical resection is beneficial regarding the survival of all patients, even those aged ≥80 years (Zinn et al., 2013; Chaichana et al., 2011; Han et al., 2020).

Clinical trials typically evaluate treatment regimens in groups of patients <65 years of age (Schmidt et al., 2020). Due to factors such as shorter life expectancy and poorer overall prognosis, as well as age-related comorbidities, older patients are often treated with less aggressive, single-modality therapies (Laigle-Donadey and Greffard, 2020). In addition, cognitive side effects from brain radiation have been shown to increase with age (Stupp et al., 2009; Sijben et al., 2008). In summary, in some cases, elderly patients cannot withstand the multimodal standard therapies of mostly six weeks due to their frailty and comorbidities (Malmstrom et al., 2012).

However, active treatment of malignant gliomas is also crucial in older people. Studies have shown that radiation and chemotherapy reduced mortality by 55%, even in elderly patients (Okada et al., 2017). This leads to the conclusion that the multimodal therapy concept should be applied regardless of age (Wick et al., 2012). When individualizing therapy for older patients, it is essential to consider performance status, individual stamina, and physical well-being (Laigle-Donadey and Greffard, 2020). In elderly patients, the therapeutic approach must be tailored to their frailty and functional status even before surgery. The performance status can also decide on a less aggressive postoperative subsequent therapy (Malmstrom et al., 2012; Laigle-Donadey and Greffard, 2020). Tools for safe surgical conditions that preserve the patients’ functionality have been studied intensively. IONM is used for electrophysiological monitoring and mapping neural structures during surgical interventions (De Witt Hamer et al., 2012). Evidence suggests that IONM may alter the risk profile of brain tumor surgery and that monitoring may improve neurooncological outcomes (De Witt Hamer et al., 2012).

Another tool for the determination of eloquent areas is the nTMS. Additionally, by nTMS-based tractography, networks are visualized, facilitating the identification of subcortical eloquent areas such as language or motor networks (Picht et al., 2013; Raffa et al., 2018).

As the elderly group is increasing, adopting the most appropriate therapy is crucial. Using preoperative brain mapping and intraoperative neuromonitoring in older people, a balance must be established between maximum tumor removal and preservation of their functional integrity.

The present study hypothesizes that pre- and intraoperative mapping and monitoring tools such as nTMS and DES combined with standard therapy for high-grade gliomas improve the neurooncological outcome and can help preserve the functional status in older people with GTR. Additionally, we aim to show that function-based decision-making toward maximal surgical resection benefits overall survival, even in older people.

## 2 Methods

### 2.1 Ethics

The study was performed in accordance with the Declaration of Helsinki and publicly registered with our university's ethics board (registration number: 2793/10, 222/14, 192/18).

Patients were only included in the study if written informed consent was given.

### 2.2 Eligibility criteria

To examine the feasibility, quality, and impact of brain mapping in the elderly, we prospectively enrolled patients with eloquent brain tumors above 65 years who underwent pre- and/or intraoperative brain mapping and were scheduled for microsurgical resection at our department between 2010 and 2020. Presumed eloquence of the tumors was defined by preoperative MRI based on the impression of eloquence by the responsible neurosurgical team and the interdisciplinary tumor board. Drawing upon established definitions of eloquence (Chang et al., 2008), we classified specific regions in our dataset as eloquent, encompassing the sensorimotor strip (precentral and postcentral gyri), language-associated perisylvian areas in the dominant hemisphere (superior temporal, inferior frontal, and inferior parietal regions), basal ganglia/internal capsule, thalamus, and the calcarine visual cortex. Patients included in the study were prospectively divided into four groups depending on their age (group 1: 65–69 years, group 2: 70–74 years, group 3 75–79 years, group 4 > 79 years). Patients without written informed consent and those with exclusion criteria for MRI or nTMS were excluded. We selected the stratification into four subcategories relying on preexisting data e.g., from Okada et al. and Ngiemphu et al. (Okada et al., 2017; Nghiemphu and Cloughesy, 2012). In terms of epidemiology, glioblastoma multiforme (GBM) predominantly manifests in individuals over the age of 65. Nevertheless, Okada et al. present findings from the Japan Brain Tumor Registry indicating that 11.4% of GBM patients were older than 75 years, with the most prevalent age cohort falling within the range of 65–69 years, constituting 17% of cases. Furthermore, investigations reveal that GBM exhibits a peak incidence among individuals aged 75–84 years, a trend anticipated to increase with the aging population (Connon et al., 2016; Nghiemphu and Cloughesy, 2012). For this reason, we divided the patients into four subgroups, each with an age range of 5 years.

### 2.3 Study protocol

Patients eligible for surgery who met the inclusion criteria underwent a corresponding MR imaging protocol (3 T MR scanner Achieva 3 T, Philips Medical System, Netherlands BV), which served as a basis for preoperative nTMS. The MRI protocol included diffusion tensor imaging (DTI) sequences with 32 orthogonal sequences for fiber tracking (DTI FT) (Negwer et al., 2017). Within 72 h after surgery, the same MRI was performed and used as a postoperative control to determine the EOR. Starting in March 2018, patients additionally received an intraoperative MRI. Surgical modalities included biopsy for tissue diagnosis or craniotomy for resection with STR with <95% and GTR with >95% EOR (Bloch et al., 2012). nTMS mappings were performed by standard protocols (Krieg et al., 2017). In short, patients were exposed to a standard object naming (ON) task. Errors included no response, hesitation, neologisms, semantic, phonological, and paraphrases. The nTMS speech mapping was generated using a standard algorithm for DTI FT by comparing the errors during and without nTMS-stimulation (Negwer et al., 2017).

In specific cases, awake glioma resection was performed using a standard asleep-awake-asleep protocol (Duffau, 2013, 2014) with cortical and subcortical electrical stimulation.

### 2.4 Clinical performance and outcome parameters

The overall survival was determined as the primary outcome parameter. Secondary outcome parameters included progression-free survival, functional and radiological outcome, morbidity and mortality, complications, and the feasibility of adjuvant therapies. A second objective was to examine the functional, cognitive, and oncological outcomes after mapping-guided brain tumor resections in those patients.

Functional postoperative performance was assessed preoperatively, postoperatively at day 5 (POD5), and after three months (POM3) using the Karnofsky Performance Scale (KPS).

The neuropathological classification was based on the most recent WHO classification for CNS tumors. Time until discharge and destination (other clinics, palliative care, rehabilitation, home, radiation) were evaluated and decisions were made in our interdisciplinary neuro-oncological board.

### 2.5 Statistical analysis

Statistical evaluation was performed using Prism 9 (GraphPad Software, San Diego, CA). Data are presented as mean ± standard deviation (SD) or median and range, depending on the data set. Statistical analyses for comparison between the two groups included Student's t-test for independent samples. In contrast, differences between more than two groups were assessed using one-way, two-way ANOVA, or a mixed linear model depending on the test requirements. Kaplan–Meier log‐rank test was conducted for survival. The accepted level of significance for all tests was P < 0.05.

## 3 Results

### 3.1 Patient characteristics

One hundred sixty-eight patients aged 65 or older treated in our clinic with eloquent gliomas were included. There was a slight male predominance in the patient cohort, with 78 (46%) female and 90 male patients (54%). The median age was 74.4 years, with the oldest patient in the study being 89 years at the time of diagnosis. The median KPS grading preoperatively was 80% (50–100%). Patients were prospectively subdivided into four groups depending on their age (Table 1), group 1: 65–69 years, 58 cases [35%]; 2: 70–74 years, 47 cases [28%]; 3: 75–79 years, 43 cases (Low et al., 2022); 4: >79 years, 20 cases [12%]). Ten patients showed WHO grade 3 (6%) tumors. All other patients (n = 158, 94%) were diagnosed with WHO grade 4 tumors.

**Table 1:** Patient characteristics.

	Total	Group 1 (65-69 y)	Group 2 (70-74 y)	Group 3 (75-79 y)	Group 4 (>79 y)	P
N	168	58	47	43	20	
Age	74.8 ± 1.7	67 ± 1.4	71.9 ± 1.4	76.9 ± 1.4	83.3 ± 2.5	
Female Sex	78 (46%)	23 (40)	21 (45%)	21 (49%)	12 (60%)	0.4
Tumor grading (%)						0.2
WHO III	6	7	11	2	0	
WHO IV	94	93%	89	98	100	
Tumor location (%)						0.8
Frontal	40	43	40	37	35	
Parietal	23	21	28	26	10	
Temporal	24	22	21	23	40	
Occipital	2	3	0	2	0	
Multiform	11	10	11	12	15	
Postop deficits (%)						0.3
No deficit	86	79.3	87	93	85	
Transient	8	10.3	6	2	15	
Permanent	6	10.3	6	5	0	
PFS (months)	5.5 (0–47)	6.8 (0–47)	6.4 (2–18)	5.7 (1–11)	2.9 (2–6)	**0.016**
OS (months)	10.5 (0–86)	11.9 (0–59)	14.2 (1–86)	10.8 (0.5–30)	4.9 (0.5–19)	**0.004**

**Fig. 1:**
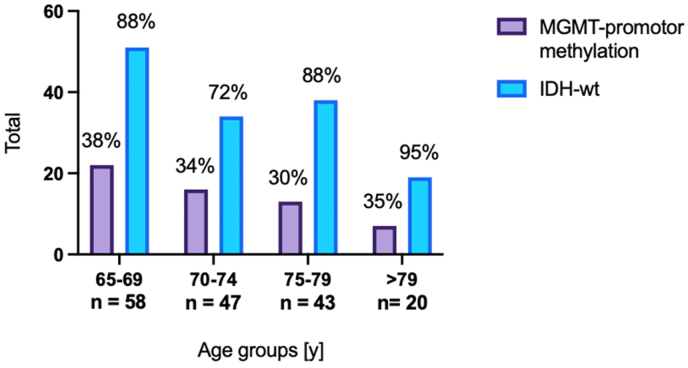
Distribution of molecular characteristics between the age groups.

58 (35%) of the patients showed an MGMT-promotor methylation. In 29 (18%) cases, the MGMT-promotor methylation status was either not specified, or the classification was not applicable. IDH mutation was found in 4 patients (2%). In 22 (13%) cases, IDH mutation was not specified nor applicable. The distribution of molecular markers between the groups is displayed in Fig. 1.

The distribution of tumor volumes between the groups was similar. However, smaller tumors tended to be treated by biopsy (GTR all groups 29.2 cm^3^ vs. Biopsy all groups 16.1 cm^3^, p < 0.05). Table 2 displays tumor volumes within the different age groups and treatment strategies.

**Table 2:** Tumor volume in cm^3^ within different age groups and treatment strategies.

		65-69 y	70-74 y	75-79 y	>79 y	All
GTR	Mean SD	28.7 27.8	29.4 21.5	25.8 21.6	32.8 30.7	29.2
	Range	0.1–111.1	0.9–77.8	4.8–86.3	5.1–100	
STR	Mean SD	35.1 31.2	28.2 30.1	24.2 20.4	44.3 16.2	32.9
	Range	1.9–116.7	1.4–104.8	1–80.3	27.2–66	
Biopsy	Mean SD	3.1 1.3	11.2 8.6	42 38.9	8.3 7.2	16.1
	Range	2–4.9	2.6–19.8	12.8–118.7	1.1–15.5	
All	Mean	22.3	22.9	30.7	28.5	

### 3.2 Treatment strategies

**Fig. 2:**
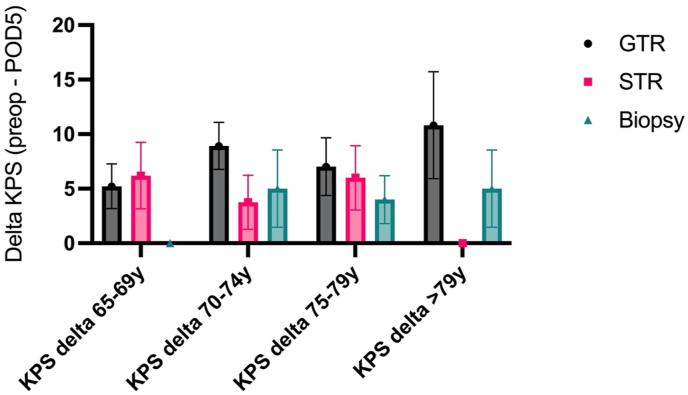
Delta KPS did not differ significantly between the age groups for each treatment strategy. There was no difference in preoperative KPS and KPS POD5 for group 1 treated with biopsy (median KPS 80, n = 3) as well as in preoperative KPS and KPS POD 5 for group 4 treated with STR (median KPS 70, n = 3), resulting in a delta KPS = 0. The higher the delta KPS, the greater the decrease in KPS at POD5 for the groups (delta KPS = preop KPS–KPS POD5).

In total, 109 patients (65%) underwent GTR, and 47 (28%) received STR. Twelve patients (7%) underwent biopsy only (Table 3). A total of 11 (7%) patients underwent awake tumor resection. The mean duration of surgery, either STR or GTR, was 200 min. Most tumors were located within the frontal lobe (62 [37%]). Table 3 shows the extent of resection amongst the different age groups. Distribution of treatment strategies did not differ significantly (p = 0.102). It is important to note that the chosen treatment strategy had no discernible effect on the KPS. Using intraoperative monitoring and selecting patients for either GTR, STR or biopsy, no significant differences in preoperative KPS and KPS at postoperative day 5 (Delta KPS) were observed across all groups (Fig. 2).

**Table 3:** EOR according to different age groups.

EOR			Group 1 (65-69 y)	Group 2 (70-74 y)	Group 3 (75-79 y)	Group 4 (>79 y)
	**Biopsy**	N	3	2	5	2
		% of group	5	4	12	10
		% of all	2	1	3	1
	**STR**	N	21	8	15	3
		% of group	36	17	35	15
		% of all	13	5	9	2
	**GTR**	N	34	37	23	15
		% of group	59	79	55	75
		% of all	20	22	14	9
**Total**		N	58	47	43	20
		% of all	35	28	25	12

Postoperatively, 100 (60%) patients received standard therapy consisting of 6 weeks of radiotherapy and concomitant temozolomide according to the Stupp protocol. Forty-three patients (26%) and ten (6%) received postoperative radiotherapy and post-operative chemotherapy only. As part of our weekly interdisciplinary neurooncological board, a decision was made on the postoperative therapy regimen depending on the final histopathology, postoperative radiological outcome, and the functional status of each patient. The group that only received the best supportive care (BSC) showed a comparably low KPS on POD5, ranging between 30 and 60 (Table 4). OS was higher in the group that received standard therapy than other treatment strategies (standard: 11.9 months, chemotherapy: 15.5 [without outlier: 6.4] months, radiotherapy: 7.3 months, BSC: 5.5 months). However, one patient underwent chemotherapy only and lived for 86 months after surgery (outlier).

**Table 4:** Treatment strategies with KPS POD5 and OS.

	Total (%)	KPS POD 5 (range)	OS (months)
All	168	60 (30–100)	10.5
Standard	100 (59.5)	70 (40–100)	11.9
Chemotherapy	10 (6)	70 (30–100)	15.5 (6.4 w/o outlier)
Radiotherapy	43 (25.6)	70 (40–100)	7.3
BSC	15 (8.9)	40 (30–60)	5.5

In the group aged >79 years, ten patients (50% of the group) received monotherapy only, either radiotherapy (40% of the group) or chemotherapy (10% within the group). The mean KPS on POD5 in the group monotherapy was decided on was 70 (60–100), whereas it was 70 (40–90) in the group of patients who received the standard therapy regimen and 30 for those treated with BSC. In summary, the older patients in our cohort more frequently underwent monotherapy instead of standard therapy based on the decision of the interdisciplinary neurooncological board. No further treatment was carried out in 9 patients (5%) due to limited functional status, and BSC was decided on. A total of 5 patients (3%) were transferred to a rehabilitation center before further therapy; all died before receiving additional treatment. One patient died during the hospital stay. Altogether, 15 (9%) patients received no other treatment and lived for a mean time of 5.5 months after surgery.

### 3.3 Pre- and intraoperative mapping characteristics

All patients underwent preoperative noninvasive mapping, intraoperative neuromonitoring in 139 cases (83%), using direct cortical and/or subcortical motor mapping in 67 (40%) and 50 (30%) cases, and awake language mapping in 12 cases (7%). 137 (82%) patients were monitored intraoperatively using MEP recordings, of which 36 (21%) had a decrease or loss of the amplitudes during surgery, with a 56% total recovery rate. 5 (14%) patients recovered 50% or more. Table 5 presents the various intraoperative modalities employed, along with the rates of decrease or loss of amplitudes and the rates of recovery across the four subgroups.

**Table 5:** Intraoperative modalities. Patients in all four subgroups underwent comparable intraoperative neuromonitoring procedures. There were no statistically significant differences observed in the incidence of amplitude loss and subsequent recovery.

	Group 1 (65-69 y)	Group 2 (70-74 y)	Group 3 (75-79 y)	Group 4 (>79 y)	P
MEP (% of group)	83	77	79	90	0.717
SEP (% of group)	31	17	26	35	0.359
TES (% of group)	72	72	56	75	0.351
DCS (% of group)	38	51	35	25	0.191
DSCS (% of group)	26	34	33	25	0.65
Decrease/loss of amplitude (% of group)	17	28	16	20	0.605
Recovery (% of all patients with decrease/loss of amplitude)	50	70	85	75	0.729
Awake Surgery (AS, % of group)	9	11	2	0	0.249
iMRI (% of group)	33	17	26	40	0.199

Of all 168 patients, 45 (27%) were monitored using SEP, and 46 (27%) underwent intraoperative MRI (iMRI). Of these, 14 patients (30%) had residual tumors resected after iMRI. KPS between patients selected for AS did not differ significantly compared to the patients who didn't receive awake mapping preoperatively (80 vs. 80). However, POD5 KPS was significantly higher in those patients who received AS (90 vs. 70, p < 0.05).

OS in the AS group was 16.8 months compared to 10.5 months in all patients, regardless of the IONM treatment strategy. In all patients, PFS in the AS group was 6.9 months and 5.5 months.

### 3.4 Functional, cognitive, and radiological outcome

**Fig. 3:**
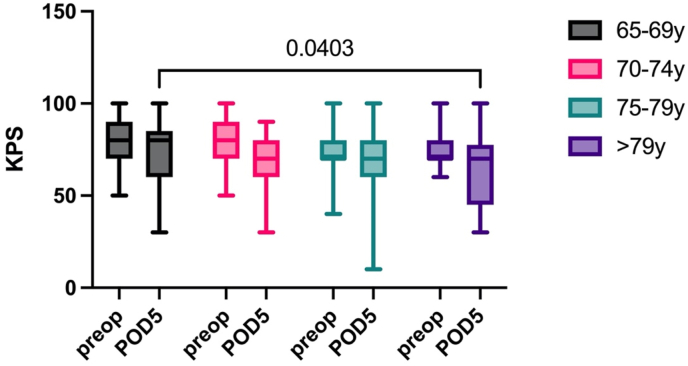
Pre- and postoperative values for KPS. The pre- and postoperative KPS did not differ between most of the groups. However, there was a significant difference seen between group 1 and group 4 on POD 5 (p = 0.04).

Eighteen (11%) patients suffered from transient motor deficits such as hemiparesis or gait ataxia, while 12 (7%) had permanent motor deficits postoperatively. Five (3%) patients suffered transient language deficits, and 10 (6%) had permanent language deficits. We found transient and permanent functional deficits in 13% and 11% of cases postoperatively without significant differences. The preoperative KPS (group 1: median 80 [range 50–100], 2: 80 [50–100], 3: 70 [40–100], 4: 70 [60–100], p = 0.25), as well as the postoperative KPS on POD 5 did not differ significantly between the first three subgroups (1: 60 [30–100], 2: 70 [40–100], 3: 70 [40–100], 4: 60 [70–90], p = 0.59, Fig. 3). However, there was a notable disparity in KPS on POD5 between group 1 and the eldest group (p = 0.04). Additionally, the surgical procedure itself exerted a significant impact on KPS, evidenced by a statistically significant decrease of KPS across all groups (p < 0.001).

### 3.5 Overall survival and progression-free survival

**Fig. 4:**
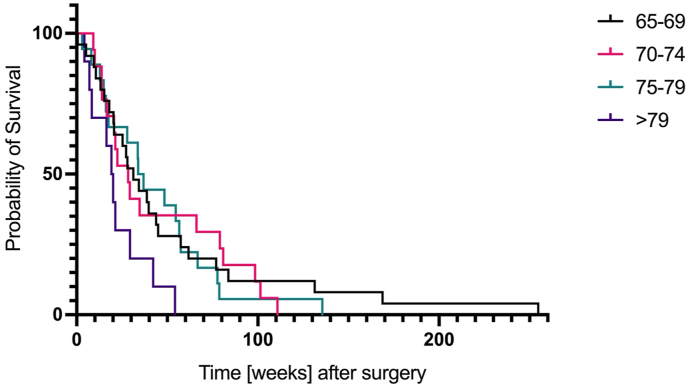
Kaplan-Meier estimation of survival probability between the different age groups. Survival was significantly different between the four groups (p = 0.004).

**Fig. 5:**
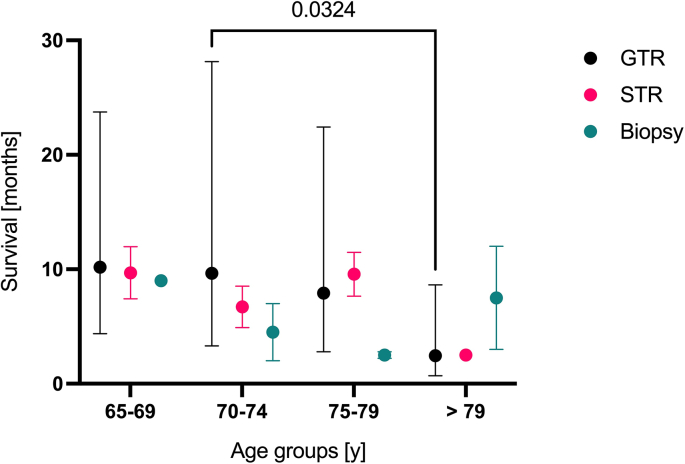
Summary data on survival in months depending on treatment strategy and age. Patients who receive GTR in group 2 have a significant longer survival than patients in group 4 (p = 0.03).

Post-operative death occurred in one (1%) case because of multi-organ failure after septic shock. With an overall long-term follow-up of 51%, patients had a progression-free survival of 5.5 (1–47) months and an overall survival of 10.5 (0–86) months. Patients receiving GTR lived significantly longer compared to the other treatment strategies, for a mean of 13.5 months (p = 0.006), while mean OS following STR was 8.9 months and following biopsy was 4.8 months (Fig. 5). Regardless of treatment, OS between the four groups differed significantly (Kaplan-Meier with log rank test, p = 0.004; Fig. 4). In addition, there was a significant difference between OS in patients with WHO grade 3 and WHO grade 4 tumors (35.5 months vs. 9.8 months, Pearsons-Chi-Quadrat test, p = 0.002).

**Fig. 6:**
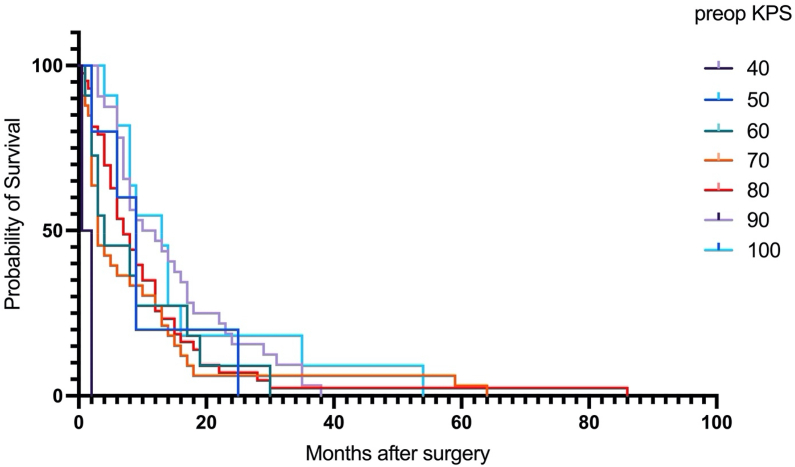
Kaplan-Meier estimates for different preoperative KPS values regardless of age. A significant difference was observed for all preoperative KPS groups (Median survival in months: KPS 40: 1.25, KPS 50: 9, KPS 60: 4, KPS 70: 3, KPS 80: 7, KPS 90: 11, KPS 100: 13, p = 0.002 log-rank).

Moreover, the preoperative Karnofsky Performance Status (KPS) exerted a notable impact on overall survival (p = 0.002). Elevated preoperative KPS, indicative of better patient functional status, was associated with increased overall survival, irrespective of age (Fig. 6).

## 4 Discussion

### 4.1 Glioma resection with preoperative brain mapping and intraoperative neuromonitoring in the elderly

We showed that the interdisciplinary treatment of older glioma patients seems to be less depending on age but must be adjusted based on the functional status of each patient. Furthermore, our findings are congruent with other studies regarding higher survival rates in patients treated with GTR compared to STR and biopsy, showing that maximum resection remains the first step of an optimal neurooncological treatment regardless of age (Klingenschmid et al., 2022; Chaichana et al., 2011). Klingenschmid et al. found that the overall survival after biopsy was shorter than after STR and GTR for elderly patients (Klingenschmid et al., 2022). These data are supported by multiple other studies, including the systematic review and meta-analysis by Almenawer et al. analyzing >12,000 older patients regarding functional recovery and tumor progression and mortality or morbidity after GTR compared to biopsy. They showed that patients who underwent GTR lived seven months longer than those receiving a biopsy, making it clear that GTR is safe and feasible. Additionally, the analysis of tumor volumes combined with outcomes of our cohort shows that even the resection of large mass lesions under focused preoperative mapping and IONM might be advantageous in older people.

However, we do not recommend that patients above 65 years should be treated with GTR, and postoperative adjuvant radio- and chemotherapy should be evaluated individually, depending on functional factors (Laigle-Donadey and Greffard, 2020). These include performance status, postoperative deficits, premorbid status, comorbidities, and life expectancy. The performance status, in our case determined by KPS, serves as an important prognostic factor to predict the outcome, including the overall survival morbidity and mortality. In the present study, the KPS analysis shows that non-invasive brain mapping allows the functional performance status. The recently published prospective study by Laigle-Donadey et al. indicated that survival in the tumor resection group of elderly patients was similar to that of the biopsy treatment group. Still, PFS, however, was significantly shorter in those patients who received tumor resection (Laigle-Donadey et al., 2022).

Furthermore, it became apparent that performance status and quality of life (QOL) were higher after tumor resection. However, it remains crucial to preoperatively evaluate the anesthetic risk and functional status and thus select the appropriate patient in this frail age group for maximal therapy. Factors that make maximal tumor resection less favorable for older people include the concern for postoperative neurological or systemic deterioration, more comorbidities, and a lower preoperative functional status. However, if the latter is at a relatively high level, maximal tumor resection is also comparably favorable for older people. In conclusion, instead of performing GTR or STR to raise the OS, the goal must be set as a reliable surgical tool to preserve the patients’ functionality. Laigle-Donadey et al. found no significant difference in survival between the treatment groups, although they did not use and analyze preoperative mapping and intraoperative monitoring techniques. In addition, the extent of resection was not disaggregated. Our data suggest that GTR provides a relevant advantage in survival and PFS over STR and biopsy.

Furthermore, the present data show that the postoperative functional status determined by KPS POD5 is a tool for deciding the adjuvant therapeutic regimen. Older patients in our cohort had a higher probability of deciding on a mono-therapeutic adjuvant treatment (chemo- or radiotherapy). Patients with a KPS POD5 around 30–40 most likely received no further therapy but were treated with BSC or discharged to a neuro-rehabilitative clinic first.

### 4.2 Limitations

Limitations include the monocentric design of our study and the fact that different treatment modalities regarding the extent of resection and histomorphology of gliomas regarding WHO grading were compared. However, there was no comparison to the outcome of patients without DES and nTMS as we had no control group. However, a historical group from pre-existing data was used for comparison. Selection bias may also be present in this study as patients with better preoperative KPS are treated more aggressively concerning the extent of resection. In addition, only the KPS and no other cognitive or functional tool were used to determine preoperative performance status.

Furthermore, KPS was determined by different investigators. Therefore, interrater variability may be a present error. Additionally, to compare older patients receiving awake language mapping, the group count of 11 may need more to generate valuable comparable data.

## 5 Conclusion

In deciding on the extent of resection, not the age but the individual preoperative performance status of each patient must be considered. Maximal resection leads to a longer overall survival and better functional outcome, as shown in this study and pre-existing data (Laigle-Donadey and Greffard, 2020; Pessina et al., 2018; Klingenschmid et al., 2022). Additionally, the balance between treatment efficacy and quality of life must be maintained due to the shorter life expectancy of older patients. The present study has shown that determining the preoperative functional status helps to balance the decision-making in glioma and glioblastoma treatment in older people. Furthermore, we were able to show that nrTMS is a safe, non-invasive, and feasible method for safe tumor resection in older people.

In summary, it is essential to conduct clinical studies in older patients, as the aging population is expanding and, especially in the case of malignant gliomas, older people are more frequently affected (Conti Nibali et al., 2021; Low et al., 2022). This and similar studies are needed to provide guidelines for the best treatment for older patients. The present findings may help guide treatment decisions in elderly patients with high-grade gliomas and glioblastomas.

## Disclosure

This research received no specific grant from funding agencies in the public, commercial, or not-for-profit sectors. This trial was funded entirely by institutional grants from the Department of Neurosurgery. The authors declare no conflicts of interest.

Sebastian Ille, Sandro M. Krieg, and Bernhard Meyer are consultants for Brainlab AG. Sebastian Ille is also a consultant for Icotec AG and Carl Zeiss Meditec AG and has past honoraria from Nexstim AG. Sandro M. Krieg is a consultant for Ulrich Medical and Need Inc. And received honoraria from Nexstim Plc, Spineart Deutschland GmbH, Medtronic, and Carl Zeiss Meditec AG. Bernhard Meyer received honoraria, consulting fees, and research grants from Medtronic, Icotec AG, and Relievant Medsystems Inc., honoraria and research grants from Ulrich Medical, honoraria and consulting fees from Spineart Deutschland GmbH and DePuy Synthes, royalties from Spineart Deutschland GmbH, and has a financial relationship with Medacta. The other authors have no personal, financial, or institutional interest in any drugs, materials, or devices described in this article.

## Conflicts of interest

This research received no specific grant from funding agencies in the public, commercial, or not-for-profit sectors. This trial was funded entirely by institutional grants from the Department of Neurosurgery. The authors declare no conflicts of interest.

Sebastian Ille, Sandro M. Krieg, and Bernhard Meyer are consultants for Brainlab AG. Sebastian Ille is also a consultant for Icotec AG and Carl Zeiss Meditec AG and has past honoraria from Nexstim AG. Sandro M. Krieg is a consultant for Ulrich Medical and Need Inc. And received honoraria from Nexstim Plc, Spineart Deutschland GmbH, Medtronic, and Carl Zeiss Meditec AG. Bernhard Meyer received honoraria, consulting fees, and research grants from Medtronic, Icotec AG, and Relievant Medsystems Inc., honoraria and research grants from Ulrich Medical, honoraria and consulting fees from Spineart Deutschland GmbH and DePuy Synthes, royalties from Spineart Deutschland GmbH, and has a financial relationship with Medacta. The other authors have no personal, financial, or institutional interest in any drugs, materials, or devices described in this article.
